# Soft and Nanostructured Materials for Energy Conversion

**DOI:** 10.3390/ma14061492

**Published:** 2021-03-18

**Authors:** Patrizia Bocchetta

**Affiliations:** Dipartimento di Ingegneria dell’Innovazione, Università del Salento, via Monteroni, 73100 Lecce, Italy; patrizia.bocchetta@unisalento.it

Soft matter is a class of materials with flexibility properties and the ability to easily deform and self-assemble into complex structures. Some examples of materials with these features are fluids, polymers, colloids, gels, and also some biological compounds. The intensity of work in the study, design, fabrication, and characterization of these materials has increased because of their fundamental role in modern devices and technologies, from biology to engineering.

On the other hand, nanostructuring of materials, to fabricate nanoparticles, nanowire, and nanotubes, for example, opens the way to novel, specific, and performant functionalities thanks to the changes in chemical bonding at the nanoscale or the combination with relevant atoms or compounds to generate composites for specific applications. Carbon nanotubes and graphene nanomaterials are great examples in terms of electric conductivity and mechanical resistance; at the same time, two-dimensional polymeric materials are able provide a larger range of possible features ranging from electric to proton conductivity, chemical functionalities and mechanical resistance, flexibility and many more. By tailoring properties, shapes, and sizes, nanomaterials have become promising performant elements in flexible energy storage and conversion devices.

This Special Issue on “Soft and Nanostructured Materials for Energy Conversion” covers the fabrication, characterization, and properties of soft and nanostructured materials for possible applications in energy conversion. This Special Issue collects five original, full-length articles on soft and nanostructured materials for energy storage and conversion [1,2,3,4,5], and one review on soft and flexible materials for power sources and sensors [6].

The first paper [1] reports on the design and development of a flexible electrochemical actuator based on an ionogel graphene composite. Electrochemical actuators have become attractive devices for smart technologies due their low driven-voltage, lightness, flexibility, and large deformation. The proposed actuator has a sandwich structure with good flexibility and is capable of delivering a high specific capacitance of about 40 F g^−1^.

The use of neutron scattering techniques to characterize nanomaterials by a direct visualization window into the electrocatalytic layers of a Polymer Electrolyte Membrane (PEM) fuel cell is proposed in another paper of the Special Issue [2]. The structural features of materials can be examined at the sub-nano to the micrometer scale with distinctive contrast variation properties, with the advantage to use materials with light elements and heterogeneous compositions. In a complementary way to electron microscopy, neutron scattering is demonstrated to be able to follow the electrode structure, made of catalyst nanoparticles and carbon, on a time and spatial scale after contact with the electrolyte. The heterogeneity of the material can be distinguished by varying the contrast and evidencing the different areas of the material. Future studies will be focused on this last aspect of the research.

Pt nanoparticles and atoms have been successfully dispersed into porous carbons to obtain efficient electrocatalysts for hydrogen evolution reactions (HER) by Kang et al. [3]. The synthesis of the nanomaterial has been carried out by the direct pyrolysis of a Pt porphyrin-based conjugated microporous polymer. The efficiency of HER showed promising mass activities two-fold greater to that of commercial Pt/C. The reasons for such results can be related to the high electric conductivity of the carbon matrix and synergistic effect of atomically dispersed Pt and ultra-fine Pt nanoparticles. The strategy proposed in the paper is also applicable to other catalytic systems for energy applications, such as oxygen reduction reactions and carbon dioxide reduction.

The functionalization of carbon nanotubes is studied by Liu et al. [4] for thermo-electric applications. Single-walled carbon nanotube (SWCNT)/Bi_2_Te_3_ composite materials are prepared by in situ reduction and subsequent cold-pressing, combining a pressure-less sintering process or a hot-pressing technique. The analysis of the thermoelectric properties of the composite material as a function of the methods of synthesis show that the hot-pressing technique gives the better result; however, the equipment requires high costs. The reason can be found in the increase in density of the bulk material: starting from this conclusion, further studies aimed to improve this physical property could enable the use of less expensive cold pressing.

The research papers of the issues are completed by the study by Pagano et al. [5], where the synthesis of ZnO nanostructures as a material able to simultaneously photo-degrade water-dissolved pollutants and enhance the photo-degradation process thanks to their piezoelectric features is investigated. The authors propose a novel cost-effective strategy to prepare hexagonal elongated ZnO microstructures in the wurtzite phase. The photo-catalysis processes activated by ZnO were successfully checked under UV-light irradiation on water-dissolved methylene blue dye. Additionally, the piezoelectric features were evaluated by depositing ZnO microstructure films onto flexible substrates and subjected to mechanical strain and UV photons. The photo-degradation efficiency strongly increased, suggesting that the process proposed in this paper allows the degradation of water-dissolved pollutants by using renewable energy sources in eco-friendly conditions.

Finally, a comprehensive review is presented which aims to be the center of the Special Issue. The review [6] focuses attention on the remarkable research contributions of the last years on soft and flexible materials used in the fabrication of electrodes, electrolytes, and substrates. In particular, the review covers the use of soft materials in fuel cells, batteries, biosensors, biofuel cells, and supercapacitors, evidencing how the soft properties allow the traditional power sources and sensors to convert to wearable electronics maintaining high power delivering. The general trend of the research literature is to use traditional materials that acquire flexibility and softness by bonding with other materials, making composites by nanostructuring, by designing performant architectures, or by using relevant assemblies of the various elements of the entire device.
